# Impacts of Seawater pH Buffering on the Larval Microbiome and Carry-Over Effects on Later-Life Disease Susceptibility in Pacific Oysters

**DOI:** 10.1128/aem.01654-22

**Published:** 2022-11-07

**Authors:** Clara L. Mackenzie, Christopher M. Pearce, Sarah Leduc, Daniel Roth, Colleen T. E. Kellogg, Rute B. G. Clemente-Carvalho, Timothy J. Green

**Affiliations:** a Fisheries and Oceans Canada, Pacific Biological Station, Nanaimo, British Columbia, Canada; b Centre for Shellfish Research, Vancouver Island University, Nanaimo, British Columbia, Canada; c Hakai Institute, Heriot Bay, British Columbia, Canada; University of Queensland

**Keywords:** *Crassostrea*, disease susceptibility, microbiome, ocean acidification, seawater pH buffering

## Abstract

Ocean acidification upwelling events and the resulting lowered aragonite saturation state of seawater have been linked to high mortality of marine bivalve larvae in hatcheries. Major oyster seed producers along North America’s west coast have mitigated impacts via seawater pH buffering (e.g., addition of soda ash). However, little consideration has been given to whether such practice may impact the larval microbiome, with potential carry-over effects on immune competency and disease susceptibility in later-life stages. To investigate possible impacts, Pacific oysters (*Crassostrea gigas*) were reared under soda ash pH buffered or ambient pH seawater conditions for the first 24 h of development. Both treatment groups were then reared under ambient pH conditions for the remainder of the developmental period. Larval microbiome, immune status (via gene expression), growth, and survival were assessed throughout the developmental period. Juveniles and adults arising from the larval run were then subjected to laboratory-based disease challenges to investigate carry-over effects. Larvae reared under buffered conditions showed an altered microbiome, which was still evident in juvenile animals. Moreover, reduced survival was observed in both juveniles and adults of the buffered group under a simulated marine heatwave and *Vibrio* exposure compared with those reared under ambient conditions. Results suggest that soda ash pH buffering during early development may compromise later-life stages under stressor conditions, and illustrate the importance of a long-view approach with regard to hatchery husbandry practices and climate change mitigation.

**IMPORTANCE** Shellfish industries are threatened worldwide by recurrent summer mortality events. Such incidences are often associated with *Vibrio* disease outbreaks, and thus, it is critical that animals are able to mount sufficient immune responses. The oyster immune system is linked to the microbiome which is laid down during early developmental stages. Consequently, shellfish hatcheries play a key role with regard to shaping the immune competency of later-life stages. This study represents the first in-depth examination of whether the adoption of seawater pH buffering practice by hatcheries for mitigation of ocean acidification may alter the larval microbiome, and thus, have repercussions for adult susceptibility to summer mortality events. Findings demonstrate that even minimal buffering results in a changed microbiome which is paralleled by increased mortality of later-life stages under *Vibrio* and temperature stressors, highlighting the importance of the hatchery environment with regard to shaping resilience to summer mortality events.

## INTRODUCTION

Current global levels of atmospheric carbon dioxide are the highest experienced in 2 millennia and continue to escalate at an unprecedented rate (1). Resulting physical changes in the marine environment, including ocean acidification (OA) and ocean warming, are well-documented (1–5) and, under current emissions scenarios, predicted to increase over the foreseeable future (1). Likewise, there is a wealth of evidence for numerous and wide-ranging impacts of climate stressors on the ecology, physiology, behavior, growth, development, and overall survival of marine animals (6–14).

In addition to background trends of warming and OA, climate change is also causing increases in the frequency and duration of acute stressor events in marine environments. These include prolonged anomalous ocean warming events (i.e., marine heatwaves) (15, 16) and upwellings during which deep (low pH) seawater migrates to coastal shallows (17–19). Along the west coast of North America, for example, upwelling events regularly deliver low-pH seawater to coastal areas (19), with such episodes often coinciding with heatwaves, hazardous algal blooms, and pathogenic bacteria (16, 20, 21).

In recent years, Pacific oyster hatcheries have suffered major losses due to the mass mortality of larvae during regional upwelling periods (14, 19). These mortality events have been attributed to low aragonite saturation levels associated with upwelled seawater, resulting in decreased availability of the carbonate isomorph (aragonite) that larval oysters rely on to build their initial shells. To counteract the impacts of upwelled seawater on larval production, hatcheries have resorted to dosing seawater with soda ash as a means of buffering low pH water (14, 19, 22). The addition of agricultural-grade soda ash (unpurified sodium carbonate, Na_2_CO_3_) results in an elevated aragonite saturation state (Ω_Ar_) (22) and the resulting return to pre-upwelling levels of larval production observed in hatcheries due to the adoption of soda ash dosing has been largely regarded as an OA mitigation success story (14, 19). However, little if any attention has been given to whether soda ash dosing may have any parallel detrimental effects on the quality (i.e., health) of larvae and on disease resistance in later-life stages.

At the same time, shellfish growers around the world continue to contend with increases in the frequency and magnitude of mass mortality events of Pacific oysters during summer months (i.e., summer mortality syndrome) (23–30). Despite extensive and concentrated research efforts, the exact causal agent(s) of summer mortality remains difficult to pinpoint. Rather, it has been described as a complex interaction of external environmental (e.g., temperature, salinity) and biological (e.g., hazardous algal blooms) factors that is further influenced by oyster age class and reproductive state (30–33). Additionally, the high temperatures associated with these events facilitate the proliferation and spread of marine pathogens (e.g., *Vibrio* spp.) thereby imposing further physiological stress on host organisms during a time of heightened environmental stress (25, 29–31, 33).

When environmental stressor conditions manifest, it is important that adequate immune responses can be maintained to combat opportunistic viral and bacterial infections. The oyster immune system is strongly linked to those communities of microorganisms inhabiting the host body (i.e., the microbiome) with which it has co-evolved (34, 35). Healthy animals typically possess microbiota that demonstrate “colonization resistance,” referring to the inhibition of colonization and overgrowth by invading microbes (e.g., pathogens) (36). Colonization resistance is associated with a stable and diverse microbiome and a controlled and reduced inflammatory response. Moreover, it plays a key role in the development of the early microbiome which then shapes lifelong immuno-competencies (34–36).

Recent investigation of the microbial communities of marine invertebrates suggests that a healthy microbiome contributes to disease resistance (37) and benefits may be prolonged over multiple generations (38). However, while it is generally agreed that alterations in microbiome composition can have direct ramifications for host immune-regulation, knowledge and understanding of the specific associations between the oyster microbiome and disease remain limited (26, 39).

The oyster microbiome has been shown to vary according to seasonal changes in microbial communities within the marine environment (40) and may be altered during stressor events (41). Likewise the microbiome of early developmental stages (e.g., larvae) is influenced by rearing conditions such as seawater quality and food source (42). It is hypothesized that soda ash pH buffering may also alter the oyster larval microbiome which could relay to reduced disease resilience in later-life stages.

The present study represents the first in-depth investigation into whether soda ash pH buffering during larval production has repercussions for later-stage oysters in terms of a diminished immune response and decreased survival. The main objectives of the work were to investigate the effects of soda ash buffering on the microbiome, immune response, and survival of larval-stage oysters, and to examine whether observed impacts relayed to later-life stages in terms of susceptibility to stressor events (e.g., *Vibrio* infection during a heatwave event).

## RESULTS

### Larval OA buffering experiment.

**FIG 1:**
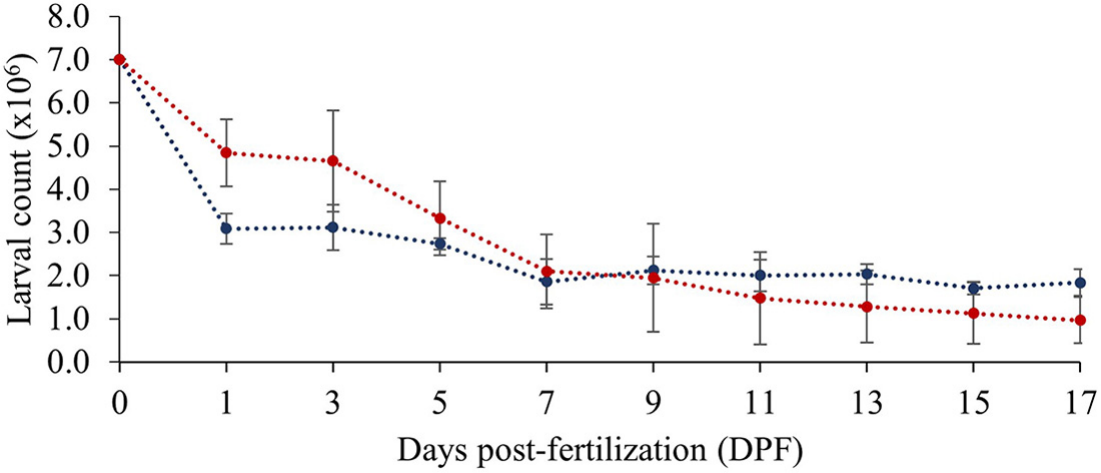
Mean ± SD larval counts under ambient (blue dashed line) and buffered (red dashed line) seawater conditions over the 0 to 17 days post-fertilization (DPF) larval developmental period.

After 24 h of development (1 day post-fertilization [DPF]), mean larval abundances in ambient and buffered seawater treatments were 3.08 ± 0.77 × 10^6^ and 4.84 ± 0.35 × 10^6^, respectively (Fig. 1). At 17 DPF, mean larval abundances had decreased in both treatments to 1.84 ± 0.31 × 10^6^ (ambient) and 0.97 ± 0.54 × 10^6^ (buffered). A two-way ANOVA showed a significant effect of time (*F*_(9, 51)_ = 42.260, *P* < 0.001) and a significant interaction between time and seawater treatment (*F*_(9, 51)_ = 2.918, *P* = 0.009) on larval abundance, but no significant effect of seawater treatment (*F*_(1, 58)_ = 0.518, *P* = 0.476). Pairwise comparisons showed no significant difference in mean larval abundance between treatments within any given day (*P* > 0.05).

After 24 h of development (1 DPF), mean shell heights in ambient and buffered seawater treatments were 68.17 ± 0.83 μm and 67.94 ± 0.79 μm, respectively. At 19 DPF, mean shell heights were 308.76 ± 7.67 μm (ambient) and 301.28 ± 6.60 μm (buffered). There was no significant effect of seawater treatment (*F*_(1, 34)_ = 0.886, *P* = 0.356) nor a significant interaction between seawater treatment and time (*F*_(5, 30)_ = 0.775, *P* = 0.557) on larval size, but there was a significant effect of time (*F*_(5, 30)_ = 620.570, *P* < 0.001).

A PERMANOVA found a significant effect of time (*pseudo-F *= 8.21, *P* = 0.001) on larval gene expression, but no significant effect of seawater treatment (*pseudo-F *= 2.40, *P* = 0.093) nor significant interaction between the two factors (*pseudo-F *= 0.87, *P* = 0.615). Visualization of gene expression data by heat map suggested increased expression of target genes at 1 DPF. Hierarchical clustering showed no grouping of samples by seawater treatment but did suggest two main clusters loosely grouped by developmental period (earlier versus later) (Fig. S2).

**FIG 2:**
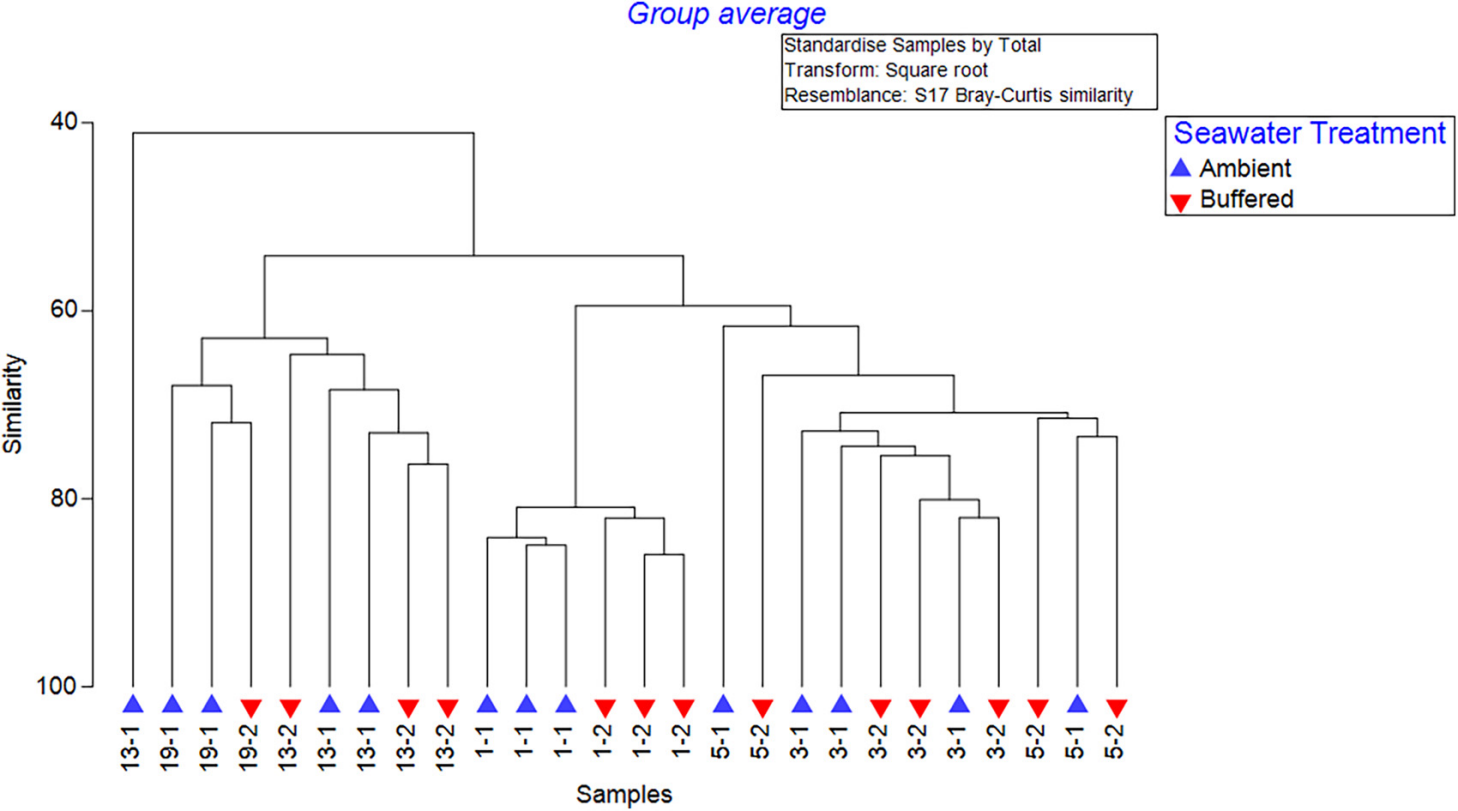
CLUSTER analyses of larval microbiome samples over the 0 to 19 days post-fertilization (DPF) larval developmental period under ambient or buffered seawater conditions. Sample coding (e.g., 1-1) indicates day of development (1, 3, 5, 13, 19 DPF) and seawater treatment (1 = ambient, 2 = buffered).

A PERMANOVA of larval 16s rRNA sequence data found a near-significant effect of seawater treatment (*pseudo-F = *1.653, *P* = 0.064) and a significant effect of time (*pseudo-F = *6.953, *P* = 0.001) on the larval microbiome but no significant interaction between factors (*pseudo-F = *1.225, *P* = 0.152). Pairwise testing identified a significant difference in larval microbiome between ambient and buffered seawater treatments at 1 DPF (*t*_(1, 4)_ = 1.509, *P* = 0.001) only. Likewise, ANOSIM suggested moderate dissimilarity between larval treatment groups (*R* = 0.314, *P* = 0.017) and strong dissimilarity between time points (*R* = 0.754, *P* = 0.001). CLUSTER analyses largely grouped samples according to time point only though did suggest a sub-grouping of 1 DPF samples into seawater treatment groups (Fig. 2).

**FIG 3:**
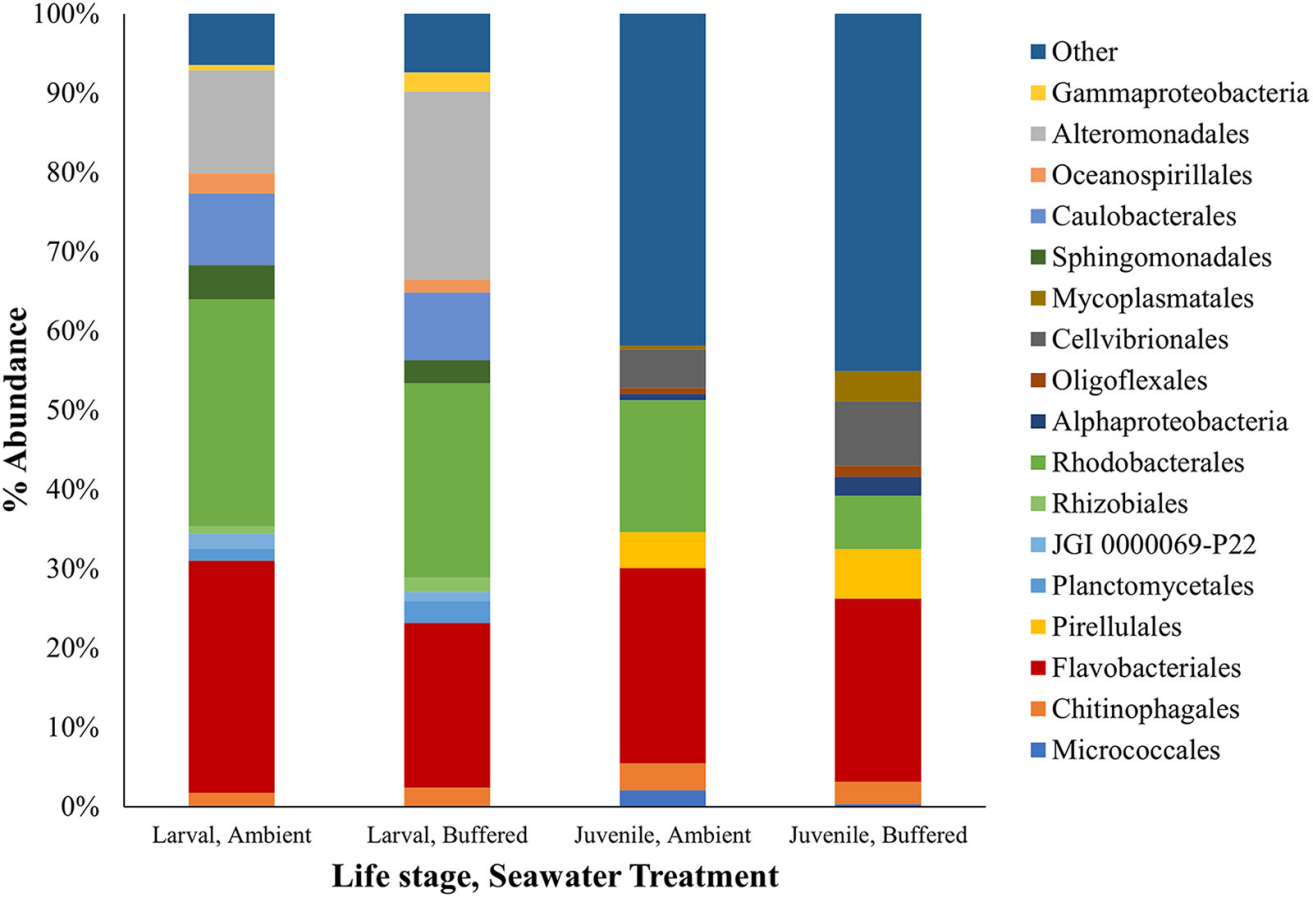
Proportional abundance (%) of the top 10 microbial groups contributing to dissimilarity in oyster larvae following 24 h of development under ambient or buffered seawater conditions and baseline (T0, prior to Vibrio aestuarianus challenge) juvenile oysters reared as larvae under ambient or buffered seawater treatment groups prior to *V. aestuarianus* challenge. All remaining microbial groups present at either life stage are shown as “Other.”

PRIMER analyses suggested that larval microbial communities varied after 24 h of development between ambient and buffered conditions. There were noticeable shifts in proportional abundance in a number of groups, including an increase in Alteromonadales and decreases in Rhodobacterales and Flavobacteriales under buffered conditions (Fig. 3). SIMPER analyses of samples at 1 DPF indicated an average dissimilarity of 19.10% between buffered and ambient groups with top contributions from Alteromonadales (7.99%), Flavobacteriales (5.18%), and Gammaproteobacteria (4.55%). Average abundances for ambient and buffered treatment groups were 3.62% and 4.89% (Alteromonadales), 5.44% and 4.62% (Flavobacteriales), and 0.82% and 1.54% (Gammaproteobacteria), respectively.

Average dissimilarity between treatments across all time points was 29.71% with top contributions from Rhodobacterales (5.24%), Flavobacteriales (3.36%), and Oceanospirillales (3.32%) with average abundances of 4.29% and 5.15%, 3.93% and 3.76%, and 2.04% and 1.22% in ambient and buffered treatment groups, respectively.

### Juvenile challenge experiment.

**FIG 4:**
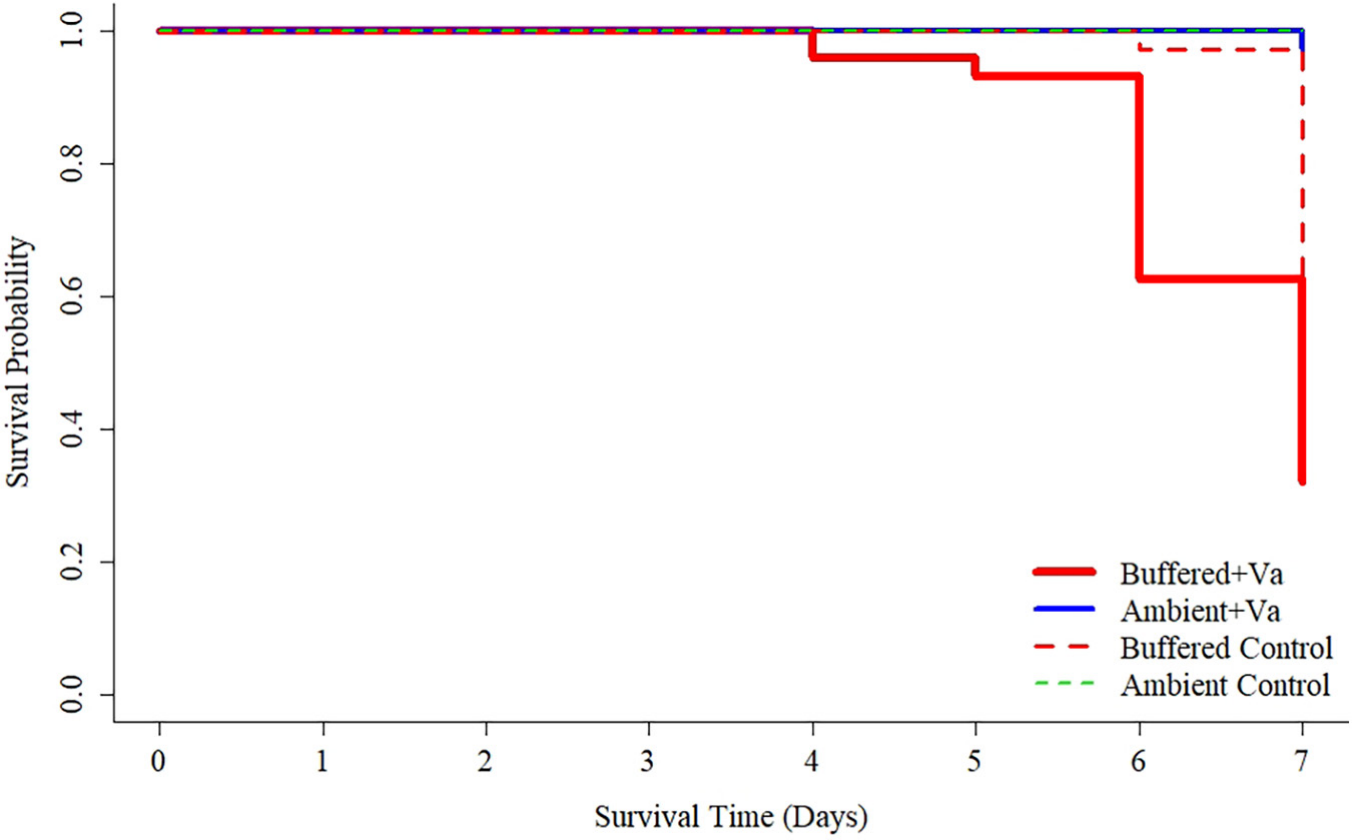
Kaplan-Meier survival curves of juvenile oysters reared as larvae under ambient (blue solid line) or buffered (red solid line) seawater conditions under a 7-day simulated heatwave and Vibrio aestuarianus exposure. Dashed lines indicate control groups not exposed to *V. aestuarianus* though are somewhat masked by the ambient treatment line.

Minimal mortality of control *C. gigas* occurred in either treatment group during the first 6 days of the week-long challenge experiment (ambient = 100% survival, buffered = 97% survival). Under *V. aestuarianus* exposure, juveniles reared under buffered conditions as larvae had increased mortality compared with those reared under ambient conditions as larvae (Fig. 4). Mortality was first noted in the buffered treatment group at day 4 with substantial declines in survival over subsequent days. In contrast, little mortality was observed in the ambient treatment group at any time point. At day 7, survival of juveniles in the buffered control declined to 65% while the ambient control maintained 100% survival. Log-rank testing showed a significant difference (χ^2^ = 35.8, df = 1, *P* < 0.001) in survival curves of buffered versus ambient treatment groups under exposure to *V. aestuarianus* (Fig. 4). Hazard analyses determined a significantly increased hazard (HR = 40.5; 95% CI = 9.8 to 167; *P* < 0.001) in the buffered group under *Vibrio* challenge in reference to the ambient group under *Vibrio* challenge.

A PERMANOVA was carried out to compare gene expression of treatment groups over the 7-day challenge experiment. Results showed a significant effect of time (*pseudo-F *= 5.966, *P* = 0.001) and *Vibrio* exposure (*pseudo-F *= 4.605, *P* = 0.011) on gene expression, but no significant effect of larval seawater treatment (*pseudo-F *= 0.667, *P* = 0.521). There were no significant interactions between any factors. Hierarchical clustering of gene expression data showed increased expression across most of the target gene set for buffered samples under *Vibrio* infection at day 5 compared with counterpart ambient samples for the same time point (Fig. S3). Hierarchical clustering subgrouped these samples but otherwise showed minimal grouping of samples according to time, larval seawater treatment, or *Vibrio* treatment.

**FIG 5:**
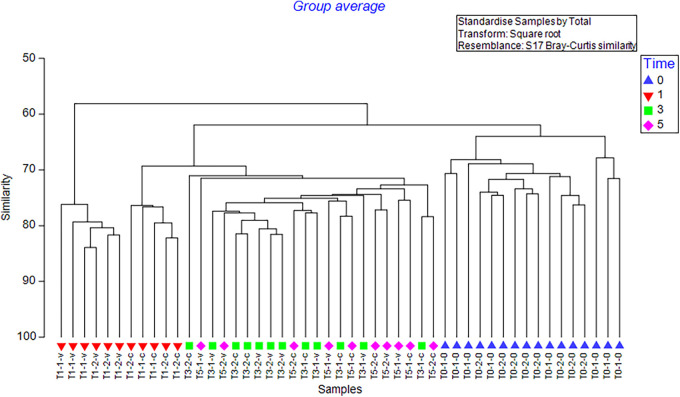
CLUSTER analyses of juvenile oyster microbiome samples under a 7-day simulated heatwave and Vibrio aestuarianus exposure. Sample coding (e.g., T1-1-v) indicates day of exposure (T0, T1, T3, T5), larval seawater treatment (1 = ambient, 2 = buffered), and challenge treatment (0 = baseline, c = control, v = *Vibrio*).

A PERMANOVA of juvenile 16s sequencing data found no significant effect of larval seawater treatment (*pseudo-F = *1.1397, *P* = 0.264) on the juvenile microbiome but did show a significant effect of time (*pseudo-F = *7.14, *P* = 0.001) and challenge treatment (*pseudo-F *= 3.437, *P* = 0.001). There was also a significant interaction between time and challenge treatment (*pseudo-F *= 2.117, *P* = 0.001). Likewise, ANOSIM suggested strong dissimilarity between time point groups (*R* = 0.75, *P* = 0.001), moderate dissimilarity between challenge treatment groups (*R* = 0.378, *P* = 0.009), and low dissimilarity between larval seawater treatment groups (*R* = 0.156, *P* = 0.009). Pairwise comparisons showed significant differences between Time points 1 and 3 (*R* = 0.884, *P* = 0.002) and Time points 1 and 5 (*R* = 1, *P* = 0.001). CLUSTER analyses generally grouped samples according to time point (Fig. 5).

PRIMER analyses showed that the microbiome varied between ambient and buffered treatment groups prior to *Vibrio* exposure (i.e., at baseline). Fig. 3 shows the proportional abundance of the top 10 microbial orders contributing to dissimilarity between the two treatment groups at baseline. SIMPER analyses of baseline samples showed an average dissimilarity between ambient and buffered treatment groups of 32.57%, with top contributions from Rhodobacterales (3.19%), Mycoplasmatales (2.18%), and Flavobacteriales (2.67%). Average abundances for ambient and buffered treatment groups were 3.93% and 2.54% (Rhodobacterales), 0.44% and 1.39% (Mycoplasmatales), and 4.76% and 4.69% (Flavobacteriales), respectively.

Average dissimilarity between treatments across all time points was 34.89% with top contributions from Vibrionales (4.03%), Mycoplasmatales (3.18%), and Flavobacteriales (2.59%). Average abundances in ambient and buffered treatment groups were 1.78% and 1.59% (Vibrionales), 1.00% and 0.96% (Mycoplasmatales), and 4.93% and 5.35% (Flavobacteriales), respectively.

### Adult challenge experiment.

**FIG 6:**
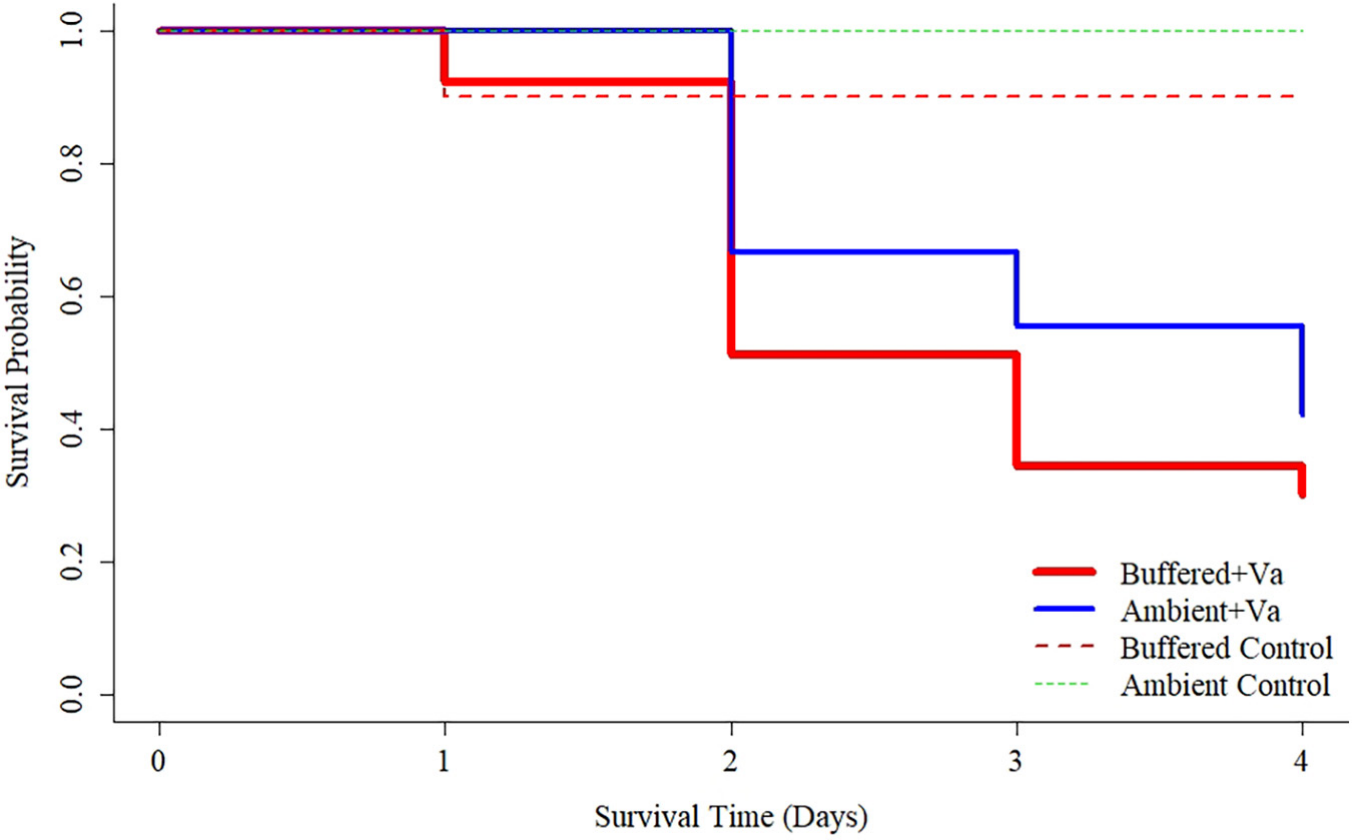
Kaplan-Meier survival curves of adult oysters reared as larvae under ambient (blue solid line) or buffered (red solid line) seawater conditions under a 4-day simulated heatwave and Vibrio aestuarianus exposure. Dashed lines indicate control groups not exposed to *V. aestuarianus*.

Minimal mortality of control *C. gigas* adults occurred during the 4-day challenge experiment (ambient = 100% survival; buffered = 90% survival). Under exposure to *V. aestuarianus*, adults reared under buffered conditions as larvae showed greater mortality across time points than those reared under ambient conditions as larvae. Log-rank testing showed no significant difference (χ^2^ = 1.5, df = 1, *P* = 0.20) in survival curves (Fig. 6) but hazard analyses determined a significantly increased hazard (HR = 1.5; 95% CI = 1.1 to 2.2; *P* < 0.025) in the buffered group under *Vibrio* challenge in reference to the ambient group under the same conditions.

## DISCUSSION

This study is the first to demonstrate that routine soda ash pH buffering in shellfish hatcheries may be partially contributing to mass mortality events of marine bivalves in later-life stages under stressor conditions. Findings highlight the importance of adopting a long-view approach with regard to husbandry and mitigation practices within hatcheries given that what may be deemed advantageous to survival and growth during bivalve larval stages may not translate to survival success over time.

Results show that even minimal (i.e., 24 h) exposure of larvae to soda ash can relay to increased mortality in juvenile and adult oysters during stressor events. Under *Vibrio* infection, the hazard ratio of juveniles reared under buffered conditions was vastly increased in reference to counterparts reared under ambient conditions. This effect was paralleled, though to a lesser magnitude, in adult animals, illustrating the long-lasting effects of short-term soda ash buffering. In fact, hatcheries are known to dose continually according to ambient seawater pH in order to achieve pre-set aragonite saturation states (i.e., 4 to 5) far beyond that which is deemed corrosive (22). Thus, it is likely that seed coming out of a number of hatcheries has experienced far greater exposure periods (e.g., all of the larval developmental period and hatchery nursery phase), with potentially greater associated impacts to immune competencies. While animals under such conditions may successfully develop to settlement, the hatchery environment is notably dissimilar to the uncontrolled and dynamic coastal environments where grow-out occurs. Hence, when juveniles are transferred to field settings where the daily maintenance, disease control measures, and optimal feeding conditions afforded by hatchery care are absent, their fitness may be strongly compromised.

Our findings also indicated no obvious benefit of soda ash buffering in terms of improved larval survival or increased size versus rearing under natural low pH conditions. Similarly, other studies have reported neutral effects (43) or even positive effects (due to local adaptation) (44) of low pH conditions during the first 24 h of development. Gimenez et al. (22) also showed that the effect of soda ash pH buffering on survival of larvae at 48-h post-fertilization varied substantially across cohorts. However, it is noted that low aragonite saturation states can cause reduced shell size and shell deformities during early development (43–45) as well as increased developmental time due to reduced ingestion rates (46). Consequently, we acknowledge that buffering of seawater may be necessary in the first 24 to 48 h of development, but maintain that alternatives to soda ash buffering should be investigated.

Larval microbiomes reported in the current study were found to be primarily composed of Alphaproteobacteria, Gammaproteobacteria, and Bacteroidetes, all of which are commonly found within marine environments (47–49). Similarly, the key contributing groups, namely, Rhodobacterales, Flavobacteriales, and Alteromonadales, are characteristic of oysters (42, 50). However, larvae reared under soda ash pH buffered conditions for the first 24 h of development showed marked shifts in proportions of Flavobacteriales (decrease), Alteromonadales (increase), and Rhodobacterales (decrease), compared with counterparts reared under ambient (low pH) conditions, with declines in Rhodobacterales and Flavobacteriales still apparent in juvenile animals.

Increases in the abundance of Alteromonadales have previously been associated with stress and disease in invertebrate species (51, 52), so the increase observed here could suggest increased larval stress and/or reduced larval health. In contrast, Rhodobacterales have been linked with disease resistance in Pacific oysters (53). Likewise, Rhodobacterales species have been shown to inhibit the growth of marine pathogenic bacteria (54) and have been applied as probiotics in hatchery environments to promote larval survival (54, 55). Consequently, the reduction in abundance of Rhodobacterales detected in both larvae and juveniles of the buffered group could potentially be related to the increased susceptibility to *Vibrio* infection witnessed at juvenile and adult stages.

Results also suggest that a change in the microbiome during the first 24 h of development may endure well beyond the larval development period. Decreased abundances of Rhodobacterales and Flavobacteriales observed in larvae were still present at the juvenile stage (i.e., ~9 months later) in animals originally reared under buffered conditions. It is therefore proposed that the observed shift in microbiome observed at larval and juvenile stages may sustain into adult stages with repercussions for altered immune competency, and thus, could be a contributory factor toward summer mortality. However, we also acknowledge that it may be simplistic to assume functional equivalence between higher level (i.e., more general) bacterial groups co-occurring across life stages and that observed parallels in microbiomes could in fact be driven by distinct species/genera.

While evidence of association between the microbiome of early developmental stage and disease susceptibility in later-life stages remains limited for invertebrate species, recent work by Fallet et al. (38) has demonstrated that the immune competencies of *C. gigas* can be improved through exposure to natural microbial communities during larval stages with improved disease resistance maintained over the subsequent generation. Similarly, various studies with mammal models have demonstrated that altered bacterial communities and/or disruption of host-commensal interactions during infancy can increase the risk of disease in later life, highlighting the role of prenatal and postnatal factors in shaping the microbiome in parallel with the immune system (56, 57).

Observed changes in the microbiome were not paralleled by significantly altered gene expression patterns between treatment groups, possibly due to only a subset of genes being included in the study or to insufficient sample numbers. Consequently, genetic biomarkers in the current study did not elucidate specific immunological mechanisms that may be impacted by the altered microbiome of buffered animals. However, prior research has demonstrated that the invertebrate microbiome does have a critical impact on host immune defenses (34, 35). For example, microbes associated with healthy marine invertebrates have been shown to aid in pathogen detection, biosynthesis of secondary metabolites, and degradation of xenobiotics (37, 58). Similarly, pathogenic microbes have been observed to manipulate host defense mechanisms (59). Specific components of invertebrate immune pathways have also been shown to play a crucial role in the recognition of both pathogenic and commensal bacteria (58). For example, the MyD88 component of the Toll-like receptor (TLR) pathway activates in response to microbial signals (60). Accordingly, the shifts in microbiome observed in buffered animals could suggest an altered ability to mount effective immune responses.

An additional consideration of pH buffering for improved aragonite saturation state is the challenge posed in terms of rearing spat that are poorly suited for natural grow-out environments where regular low-pH upwelling events (and other stressor events) are becoming customary (17, 19). Recent work has demonstrated a strong genetic basis for larval resilience to OA via development of OA-resilient lineages using pedigreed family lines (45, 61–63). Thus, an arguably enhanced strategy for ensuring survival through larval stages without compromising adult health and survival is the development of selective breeding programs for improved resilience to environmental stressors.

We propose that the purported benefits of soda ash pH buffering for mitigation of OA impacts to shellfish larvae are short-lived and, in fact, may cause greater harm to the health and ultimate survival of these animals. In light of the continued threat of summer mortality and climate change, a more apt route is to use the hatchery environment to promote and develop the health and fitness of shellfish so that they may thrive in natural field settings. Findings presented here will inform the wider research theme of climate change resilience and provide insight into the potential complexities that may be associated with climate change mitigation.

## MATERIALS AND METHODS

### Larval OA buffering experiment.

Adult (~50 mm shell height) *C. gigas* broodstock were sourced in August 2020 from a shellfish producer (Stellar Bay Shellfish, Ltd.) based in Baynes Sound, British Columbia (BC), Canada. Baynes Sound is located within the Salish Sea, a region characterized by high levels of total dissolved inorganic carbon (TCO_2_) due to organic matter remineralization and restricted connectivity to the open continental shelf. Low ratios of total alkalinity to TCO_2_ result in a reduced seawater buffering capacity and the region’s water column has corrosive aragonite saturation states and decreased pH (~7.7 to 7.8) conditions at depth (>20 m) throughout the year, which extend to surface layers throughout the winter months (17). Animals were transported to Vancouver Island University’s (VIU) Deep Bay Marine Field Station (DBMFS) located near Baynes Sound and spawned within 24 h of collection.

On the day of spawning, six high-density larval rearing tanks (cylindrical, ~250 L) were filled with ambient (i.e., natural seawater pumped directly from Baynes Sound) 5 μM-filtered seawater and held at ~21 to 22°C. Tanks were filled with seawater just prior to spawning, allowing for the preservation of naturally occurring low aragonite saturation levels associated with Baynes Sound (17). Technical grade soda ash (Univar Solutions, Redmond, WA, USA: 80 to 100% purity, batch variation) was added to half of the tanks and mixed via gentle stirring until pH_NIST_ values of ~8.0 to 8.1 were achieved. Prior to experimentation, pilot tests carried out with molecular grade (Sigma-Aldrich Canada Co., Oakville, ON, Canada: S7795, >99% purity) and technical grade soda ash varieties showed no difference in effect with regard to larval survival and size (Fig. S1). All tanks receiving soda ash (*n* = 3) are henceforth referred to as the buffered treatment while control tanks without soda ash (*n* = 3) are referred to as the ambient treatment.

Oysters were strip-spawned and crossed to produce three genetic bi-parental families. Fertilization was confirmed by the presence of polar bodies. Each family was split across two tanks (7 million eggs tank^−1^) covering the two experimental treatments (i.e., 50% reared under ambient conditions, 50% reared under buffered conditions) to rule out genetic effects. Tanks were left static for 24 h with gentle aeration to maintain the eggs in the water column. At 1 DPF, tanks were drained and larvae retained on 48 μM screens. Following tank cleaning, larvae were returned to their original tanks and placed on flow-through with ambient seawater (~24 to 25°C). Tank cleaning was carried out every 2 days until larval settlement. Temperature, pH, dissolved oxygen, and salinity of all tanks were monitored twice daily using a YSI multiprobe (Pro-DSS, YSI Incorporated, Yellow Springs, OH, USA). At 19 DPF, settlement was induced via emersion in epinephrine (0.15 g, 0.0008 M) (Sigma-Aldrich Canada Co., Oakville, ON, Canada: E4375) made up in seawater, and all spat were moved to the DBMFS nursery system (ambient flow-through seawater system) for grow-out. Animals were fed mixed culture algae (e.g., *Tisochrysis* [T-Iso], *Chaetoceros*, *Tetraselmis*, *Pavlova*) on a daily basis until deployment in the field.

Every 2 days during the larval development period, triplicate counts (3 × 1 mL) were conducted and triplicate larval samples (3 × 1 mL) were taken across all tanks. Samples were preserved at −80°C for gene expression and microbiome analyses or in 10% neutral buffered formalin (NBF) for sizing analyses. Size (shell height) was determined using a compound microscope with ocular micrometer (N = 30 per replicate tank per time point).

Seawater was sampled from each tank (*n* = 3 per treatment) on spawning day, at 1 DPF, and every 2 days until settlement. Samples were collected in amber glass bottles (340 mL), fixed via the addition of supersaturated mercuric chloride (Sigma-Aldrich Canada Co.: #413445), and sealed. The pH and salinity of seawater samples were measured with Multilab IDS 4110-3 pH and Multilab IDS 4310 conductivity sensors (YSI Inc.), respectively, calibrated with NIST buffers (Thermo Fisher Scientific Company, Hampton, NH, USA). Total alkalinity was measured using an Orion Star T910 pH Titrator (Thermo Fisher Scientific Company) with an HCl titrant (Sigma-Aldrich Canada Co.: H1758) standardized against Trizma base (Sigma-Aldrich Canada Co.: T6791) and certified seawater reference material (Dickson: Batch #174). Seawater carbonate chemistry parameters were calculated using CO2Sys (64). Table 1 shows parameters at 0 and 24 h. Due to larval tanks being gentled aerated (~400 ppm CO_2_) for the first 24 h of development, as is typical hatchery practice, the pH of ambient tanks rose from 7.63 (±0.03) to 7.96 (±0.02) over the first day. For the remainder of the developmental period there was minimal variation in carbonate chemistry parameters across replicate tanks/treatments.

**TABLE 1:** Seawater carbonate chemistry values for ambient and buffered treatments of the larval ocean acidification buffering experiment at 0 h and 24 h^*a*^

Treatment	Time	Temp (°C)	Salinity (ppt)	pH_T_^*b*^	TA (μmol L^−1^)	*p*CO_2_ (ppmv)	Ω_arag_
Ambient	0 h	21.28 ± 0.21	28.80 ± 0.10	7.63 ± 0.03	2099.19 ± 10.47	1272.37 ± 79.34	1.14 ± 0.07
	24 h	22.70 ± 0.70	29.08 ± 0.10	7.96 ± 0.02	2186.61 ± 80.40	539.10 ± 27.293	2.43 ± 0.14
Buffered	0 h	20.43 ± 0.13	28.98 ± 0.49	8.09 ± 0.00	2441.03 ± 4.64	472.16 ± 2.56	3.22 ± 0.02
	24 h	21.48 ± 0.10	29.05 ± 0.06	8.10 ± 0.01	2347.85 ± 14.34	415.38 ± 6.340	3.29 ± 0.06

a Means ± SD shown for all parameters.

b pHT, pH on total scale; TA, total alkalinity; *p*CO_2_, partial pressure of carbon dioxide; Ω_arag_, aragonite saturation state.

### Juvenile challenge experiment.

Juveniles (~5 to 10 mm shell height) were exposed to a 7-day simulated marine heatwave and *Vibrio* challenge 3 months post-settlement to investigate any carry-over effects of soda ash buffering on immune response and survival in juvenile *C. gigas*. Vibrio aestuarianus was selected for the challenge as it has been shown to contribute to mass mortalities of adult *C. gigas* within the study area (Baynes Sound) (65) and in European oyster populations (66). Vibrio aestuarianus was grown overnight with constant agitation at 21°C in tryptic soy broth containing 2% NaCl (TSB + 2% NaCl) with inocula taken from frozen glycerol stocks and tested for purity prior to use. Following, cells were washed and resuspended to OD_600_ = 1.9 with concentration based on pilot studies carried out using subsets of individuals from each treatment group. The relationship between OD and CFU was determined by serial dilution plating with an OD of 1.9 equaling approximately 2.22 × 10^8^ CFU mL^−1^.

Twelve juveniles from each replicate (*n* = 3) of the original ambient and buffered treatment groups were added to each of five 15-mL petri dishes per challenge treatment group (control, *Vibrio*) for mortality assessment (duplicate) and genetic (gene expression, microbiome sequencing) analyses (triplicate). Twelve mL of 5-μM filtered seawater were added to all petri dishes. Following, 1 mL of *V. aestuarianus* culture solution or autoclaved seawater was added to the *Vibrio* treatment group and control group petri dishes, respectively. An additional 150 μL of live algal culture (50% *Tetraselmis*, 50% *Isochrysis*) was added to each dish to encourage take-up of *Vibrio*. Following, animals were fed 150 μL of microalgae on a daily basis. All containers were incubated at 24°C for 7 days and mortality was assessed daily. Any animal gaping after a tactile stimulus with sterile forceps was deemed deceased or moribund and removed. Additionally, one animal was removed from those petri dishes designated for genetic analyses (N = 3 per replicate) on a daily basis.

### Adult challenge experiment.

Approximately 9 months post-settlement, a second simulated marine heatwave and *Vibrio* challenge experiment was carried out to investigate carry-over effects of soda ash buffering to immune response and survival in adult *C. gigas*. Prior to the experiment, juveniles arising from the initial larval OA buffering experiment were deployed in lantern nets (suspended from a raft) for grow-out in the field at Deep Bay (DBMFS culture site).

Before the challenge, all animals were notched on the distal portion of the shell (adjacent to the adductor muscle) for later inoculation. Following, 12 haphazardly selected animals from each replicate (*n* = 3) of the original ambient and buffered treatment groups were placed in each of four 20-L glass tanks with one tank acting as a control and three acting as replicates for a *V. aestuarianus* treatment group. Seven liters of 5-μM filtered seawater were added to each tank and animals were kept overnight in a temperature controlled room at 16°C with constant air supply via an air stone set up in each tank. The following day, any mortalities (N = 7 across all tanks) were removed and numbers per tank were reduced to 10 across all tanks. All *Vibrio* treatment animals were then inoculated with 50 μL of *V. aestuarianus* solution via injection into the adductor muscle. Vibrio aestuarianus was cultured as previously described (OD_600_ = 2.4 per 50 μL dose; CFU = 2.80 × 10^8^ mL^−1^). All control animals were injected with 50 μL of autoclaved seawater. Seawater temperature was then raised to 24°C and mortality was assessed over the next 4 days according to the criteria previously described.

### Genetics.

**(i) Nucleic acid extraction.** Genomic DNA and total RNA were extracted from separate samples for larval analyses and co-extracted from a single sample for juvenile analyses. For the latter, whole soft tissues were homogenized in lysis buffer and then divided for RNA/DNA extraction. All larval and juvenile RNA extractions were carried out according to TRIzol LS Reagent (Thermo Fisher Scientific, #10296028) and TRIzol Reagent (Thermo Fisher Scientific, #15596026) protocols, respectively. Total RNA for all samples was reverse transcribed using iScript Select cDNA Synthesis kits (Bio-Rad Laboratories, Hercules, CA, USA: #1708896) with random primers (10 μL reaction volume). All DNA extractions were carried out using DNeasy Blood and Tissue kits (Qiagen, Hilden, Germany: #69504).

**(ii) Transcriptomics.** Genes associated with early shell development (45) and bivalve immune responses (67–69) were selected to examine potential impacts of seawater buffering to larval shell development and immune modulation in both larvae and juveniles (Table 2). mRNA expression levels of target and internal reference genes (EFU) were determined for larval samples at 1, 3, 5, 7, 9, 11, 13, 15, 17, and 19 DPF (*n* = 3 per treatment group per time point) and juvenile samples at 1, 3, and 5 days post-inoculation (*n* = 3 per treatment group per time point). RT-qPCR was carried out with 10-μL reaction volumes comprised of 5 μL of SsoAdvanced Universal Sybr green Supermix (Bio-Rad Laboratories: #1725270), 0.5 μL of gene-specific primers, and approximately 100 ng of cDNA. All amplification reactions were run in a Bio-Rad CFX384 Touch Real-Time PCR Detection System with an initial denaturation (95°C, 2 min) followed by 40 cycles of denaturation (95°C, 10 s) and hybridization-elongation (60°C, 30 s).

**TABLE 2:** Primer pairs for genes associated with (a) larval shell development and (b) immune function in the Pacific oyster (*Crassostrea gigas*) used for RT-qPCR expression analyses

Gene	GenBank ID	Forward primer	Reverse primer
(a) Larval shell development			
Nacrein 1 (Na1)	XM_034475244	ACCAGTGTCTCCACCTACAG	ACAAGCCTACAGCCTCGCTC
Nacrein 3 (Na3)	NM_001305306	GACCATCCAGACTTGGAC	CTCTGGTCACAAGGGAGAAC
Nacrein 5 (Na5)	NM_001308915	CTACACCTACCCAGGATCTC	CTTCAACACGCTGCAATGC
pif-177	XM_011458096	CTACCACTGTGCCTTACTC	CGTAATCAACGTTAGACAGC
slc4	XM_011441189	CATCATCCAGGTCCTCTGTC	CAGCGAAGCTCTCTCTGC
nka	XM_011436470	ACATCGGACCCATTGAGTAC	CTGGTTGTGCCGGATGTTC
Tyrosinase (Tyr)	NM_001305292.1	CTTCGTACGATTCTTGTGGTC	CAGACTGACTGATATGGCTTC
(b) Immune response			
Tumor necrosis factor (TNF)	CU996035	GGATACGCAAGAGGAACTGC	TGGACATTAACGACACGCGC
Galectin (GAL)	FP002934	ACGAAACGCTCTGATTGGTG	TTAGTGGCATGGTAGGTCTG
CDC42	CU997513	AGTGACGTCTTTCTAGTGTG	GAACCTGAATATCTGGTTGC
Heat shock protein 20 (HSP20)	MT737796.1	CCGAAGGAAGAGGACCAGGAGATG	CGAACACCGACAGGTCTAAACTCTC
Heat shock protein 70 (HSP70)	AF144646.1	AACGGTATCCTGAATGTGTC	CTTCTCGTCTTCCTGCTTG
Toll-like receptor 7 (TLR-7)	KC700619.1	CTCACCAATTTGAACCTTGC	GCAGTTTTTGTAGGCTGACC
Transforming growth factor (TGF)	AM859022	CGGAGGAGTTCAAACAACTG	TCGAGTAACGTAGTCGTTGG
Myeloid differentiation 88 (MyD88)	EKC40070	GTGACTACACCAAGCAGGAC	GTACTGACCCTGAGTTCTGC
Interferon-induced protein 44 (IFI44)	CU999601	AAGATCCAACGATGAAAGAC	TTGTCGACATCACTACAAAC
Transcription factor Rel 1 (Rel1)	CU996382	GCTGAACCAGAACCTCATGA	CGAAGGACATGTTCTGATCC
Inhibitor of NF-kappa B mRNA (IkB1)	HQ650768	GCTCGGAAGTAAATGAAGTG	CTGGAGTTCTTGAGGTCTGC
Inhibitor protein kappa B mRNA (IkB2)	DQ250326	GAAAAAGTGGCAAGAGTGTC	GAAGAGTCATCGAAAGCAAC
Interleukin 17 (ILI7)	FQ664877	ACTGAGGCTCGATGCAAGTG	AGCCTTCTTGCTTCATGTGG
MPEG	EF627979	GCCACCGAAAGCCGGAGAAGATGTC	ACCGAGACCGAGTTTCAGGGGGTAG

**(iii) 16S amplicon sequencing library preparation.** 16S rRNA gene amplicon sequencing (targeting 16S V3 and V4 regions) of larval DNA samples at 1, 3, 5, 13, and 19 DPF and juvenile DNA samples at time points 0, 1, 3, and 5 days post-inoculation was carried out according to the 16S Metagenomic Sequencing Library Preparation manual (Illumina Inc., San Diego, CA, USA: #15044223) with some modifications, as follows. Amplicon PCR with overhang adapters was carried out with an iProof High-Fidelity PCR kit (Bio-Rad Laboratories: #1725330) in 20-μL reaction volumes comprised of 10 μL of iProof HF Master Mix,10 ng of gDNA, 6 μL of molecular grade water, and 1 μL of 10 μM 16S primers. Index PCR was carried out using an iProof High-Fidelity PCR kit (Bio-Rad Laboratories: #1725330) in 40-μL reaction volumes comprised of 20 μL of iProof HF Master Mix, 5 μL of gDNA, 5 μL of molecular grade water, and 5 μL of Nextera XT Index Primers. All PCR cleanup was carried out using QIAquick PCR Purification kits (Qiagen: #28104). Larval and juvenile 16S amplicon libraries were quantified using the NEBNext Library Quant kit for Illumina (Bio-Rad Laboratories: E7630). Indexed samples were diluted to 4 nM and pooled prior to sequencing.

**(iv) Sequencing.** Larval and juvenile 16S metagenomic libraries were outsourced to the Hakai Institute (Marna Genome Lab), Quadra Island, BC and sequenced using the Illumina MiSeq platform (Illumina Biotechnology Company, San Diego, CA, USA) (MiSeq v. 3 reagent kit 2 × 300 bp PE). A 60-μL PhiX DNA spike-in control was added to improve data quality. Illumina sequence data were processed using DADA2 (70) within the QIIME 2 pipeline v2019.4 (71). In brief, reads were demultiplexed and denoised prior to merging and assignment into actual sequence variants (ASVs). Taxonomic classification of ASVs was performed using the SILVA reference taxonomy v132 (72) with a custom trained classifier (73), and mitochondrial and chloroplast sequences were removed. All samples were rarefied to 20,000 sequences per sample and ASVs representing less than 0.05% relative abundance in an individual sample were filtered from the data set. Level 4 (order) ASV abundance data were then imported into PRIMER v7 +PERMANOVA for multivariate analyses.

### Analyses.

Two-way ANOVAs were carried out in RStudio (version 1.4.1106) (74) to examine the effect of larval rearing conditions, time, and their interaction on larval size and abundance. Where significant differences were detected, Tukey’s HSD method for multiple comparisons was applied. Data were tested to ensure assumptions of normality (Kolmogorov-Smirnov test, *P* < 0.05) and homogeneity of variances (Levene’s test, *P* < 0.05) were met prior to analyses. Means ± SDs are presented.

The effect of seawater treatment on gene expression was tested via permutational analysis of variance (PERMANOVA) using PRIMER V.7 + PERMANOVA (Plymouth Marine Laboratory, UK). Relative gene expression data were square-root transformed and similarity matrices prepared using Bray-Curtis distance with 999 permutations of residuals performed under a full model. Additionally, hierarchical clustering was performed using Multiple Array Viewer software (MeV v4.8.1). Data were z-transformed prior to carrying out average linkage clustering with Spearman Rank Correlation as distance metric.

Multivariate analyses of larval and juvenile microbiome abundance data were carried out using PRIMER V.7 + PERMANOVA to examine the role of larval water treatment and/or challenge treatment on bacterial abundance across time points. Standardized data were square-root transformed and resemblance matrices prepared using a Bray-Curtis distance with 999 permutations of residuals performed under full models. PERMANOVAs were conducted to examine the main effects of time, treatment, and their interaction with subsequent pairwise tests performed on significant interaction terms (*P* < 0.05). ANOSIM analyses were also carried out to examine the scale of effect (*P* < 0.05). CLUSTER and SIMPER analyses were then applied to visualize differences/similarities among groups and to identify the key bacterial orders driving the main differences among treatment groups, respectively.

Kaplan-Meier survival curves of juvenile and adult oysters were generated in RStudio (version 1.4.1106) and compared via log-rank testing. Cox proportionate hazards analyses were carried out to investigate associations between survival time and predictor variables.

### Data availability.

Sequencing data sets generated during and/or analyzed during the current study are available in the National Center for Biotechnology Information (NCBI) repository. Other data sets generated during and/or analyzed during the current study are available from the corresponding author on reasonable request.
