# Supplementary material for: Impacts of Seawater pH Buffering on the Larval Microbiome and Carry-Over Effects on Later-Life Disease Susceptibility in Pacific Oysters
Source: Appl Environ Microbiol. 2022 Nov 7;88(22):e01654-22. doi: 10.1128/aem.01654-22 (PMC9680617; doi:10.1128/aem.01654-22)
Supplement: Supplemental file 1 — Supplemental material. Download aem.01654-22-s0001.pdf, PDF file, 0.1 MB [file aem.01654-22-s0001.pdf]

Supplementary Figures

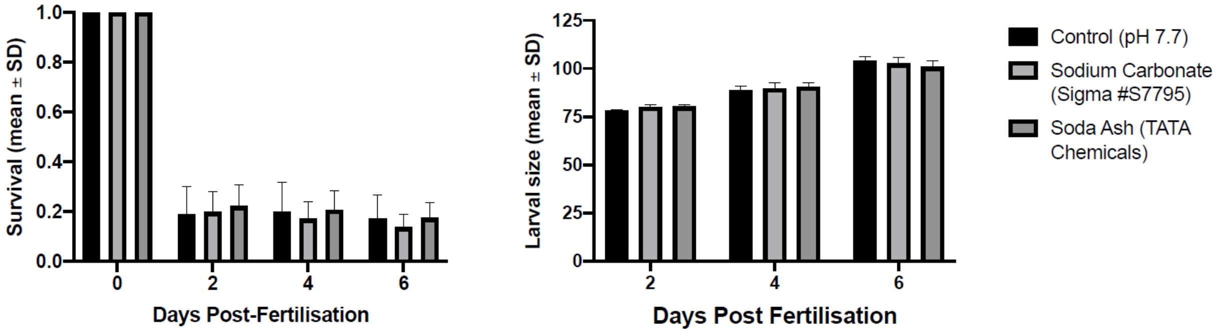

Supplementary Figure 1. Results of pilot study comparing effects of technical grade versus molecular grade soda ash varieties on larval survival and size.

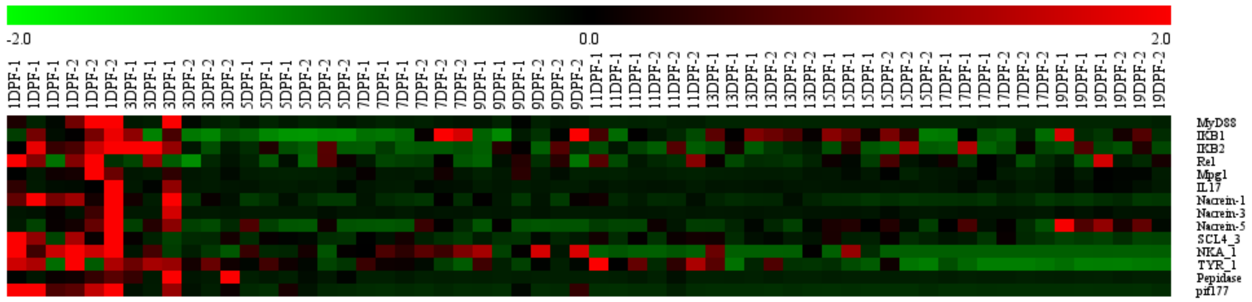

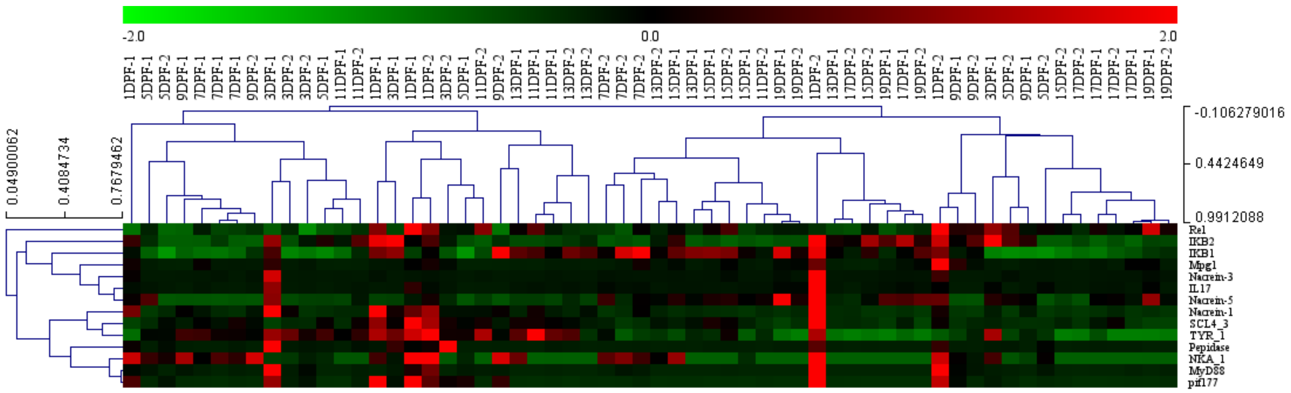

Supplementary Figure 2. (a) Heat map and (b) hierarchal clustering analyses for larval gene expression data (z-transformed). Coding (*e.g.* 1DPF-1) indicates day of development (1-19 DPF) and seawater treatment (1=ambient, 2=buffered). Replication is n=3 per day per treatment. Genes of interest are provided along the right side of the heat map.

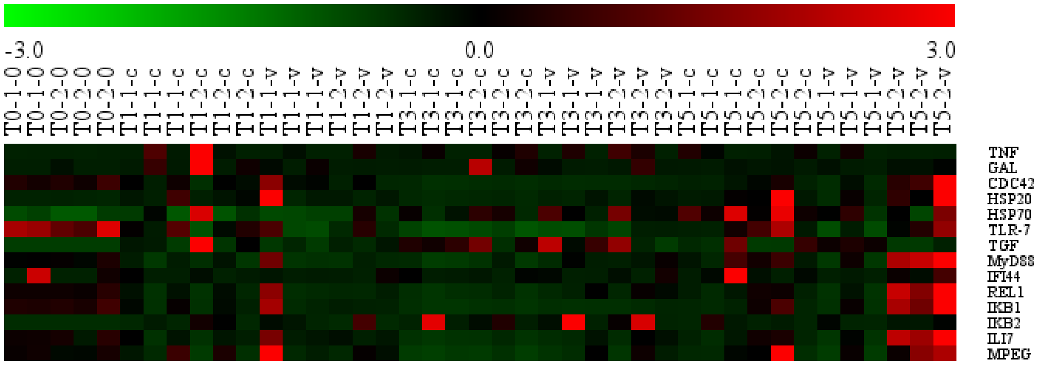

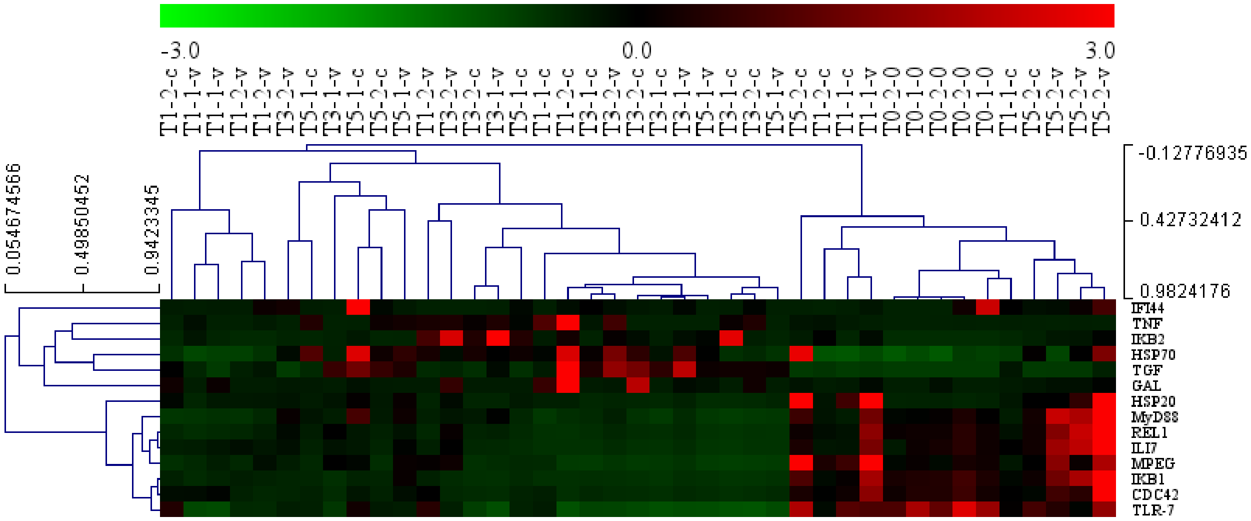

Supplementary Figure 3. (a) Heat map and (b) hierarchal clustering analyses for juvenile gene expression (z-transformed) under *V. aestuarianus* infection. Coding (*e.g.* T1-2-c) indicates day of exposure (T0, T1, T3, T5), larval seawater treatment (1=ambient, 2=buffered), and challenge treatment (0=baseline, c=control, v=*Vibrio*). Replication is n=3 per day and treatment except for T0-1-0 (n=2). Genes of interest are provided along the right side of the heat map.
